# Virtual Reality in Clinical Practice and Research: Viewpoint on Novel Applications for Nursing

**DOI:** 10.2196/34036

**Published:** 2022-03-16

**Authors:** Hyojin Son, Alyson Ross, Elizabeth Mendoza-Tirado, Lena Jumin Lee

**Affiliations:** 1 Translational Biobehavioral and Health Disparities Branch National Institutes of Health Clinical Center Bethesda, MD United States

**Keywords:** virtual reality, health care, application, nursing

## Abstract

Virtual reality is a novel technology that provides users with an immersive experience in 3D virtual environments. The use of virtual reality is expanding in the medical and nursing settings to support treatment and promote wellness. Nursing has primarily used virtual reality for nursing education, but nurses might incorporate this technology into clinical practice to enhance treatment experience of patients and caregivers. Thus, it is important for nurses to understand what virtual reality and its features are, how this technology has been used in the health care field, and what future efforts are needed in practice and research for this technology to benefit nursing. In this article, we provide a brief orientation to virtual reality, describe the current application of this technology in multiple clinical scenarios, and present implications for future clinical practice and research in nursing.

## Introduction

Virtual reality (VR) is a type of extended reality technology that is increasingly being used in health care, from assisting medical staff in practicing new techniques to supporting treatment procedures and wellness activities [1-4]. In nursing, VR technology has been primarily used in nursing education, such as simulation-based skills training and distance learning [3]. Although there is growing evidence that VR can be used in more innovative ways, such as improving troublesome physical and psychological symptoms [5,6], many nurses in clinical practice and research may be unfamiliar with novel applications of VR technology. In this article, we provide a brief orientation to VR, describe the current application of this technology in health care, and present implications for clinical practice and future research in nursing.

## A Brief Look at Virtual Reality

VR is defined as “an artificial world made up of computer-generated images and sounds and is influenced by the actions of an individual who is experiencing that world” [7]. The technology stimulates a user’s multiple senses, enabling the user to interact with realistic 3D virtual environments [5,8]. VR is different from augmented reality, which is another type of extended reality technology; while augmented reality superimposes digital data onto the real world, VR shuts out the real world and provides interaction with the simulated virtual world [9]. Simulated virtual worlds can be delivered in a nonimmersive or immersive manner. Nonimmersive VR implements the virtual environment by projecting it onto a large display or wall screen (eg, Powerwall screens and cave automatic virtual environments), while immersive VR commonly applies a head-mounted display to provide full immersion and interaction with the virtual environment [10].

VR technology has become more immersive, affordable, and portable due to the ubiquity of mobile high-performance computing and the availability of various software programs [8]. VR is being used in various fields such as education, business, medical and military training, treatment of mental health and traumatic disorders, and entertainment [4]. In particular, the use of VR has increased for recreational purposes during the COVID-19 pandemic, with users reporting its positive impact on physical activity and mental well-being [4]. In recent years, VR technology has also been recognized for its value of application for health-related purposes, including symptom improvement and pain management in diverse populations [5,11]. As VR continues to advance, we expect more active use of the technology in health care practice and research.

## Virtual Reality in Health Care

Features of VR have gained attention in the health care field, and in particular, this technology has been actively used in education and training for students and staff in medical professions. In medical education, VR has been often used for surgical and physical examination skills training, acquisition of anatomical knowledge, and building empathy for patients with neurodegenerative diseases [1]. Although VR has a comparatively short history in nursing, VR has been mainly used for nursing education purposes to optimize nursing students and nurses’ acquisition of practical skills and theoretical knowledge. Some examples include urinary catheterization practice, basic life support training, and communication practice with patients having virtual dementia [3]. Most participants who experienced VR in medical and nursing education indicated that VR-based trainings were motivating and helpful in acquiring knowledge and skills [1,3]. With the high demand for contactless learning due to the recent COVID-19 pandemic, the application of VR to health care education has never been more prominent.

The application of VR is not limited to these education or training purposes but is increasingly being used for therapeutic purposes as well. Several major therapeutic purposes of VR use include distraction from pain or uncomfortable medical procedures [2,11], relaxation and mindfulness for stress and symptom improvement [12-15], cognitive coping as psychotherapy [16,17], and rehabilitation of neuromotor functions [18]. VR has shown promising levels of effectiveness in various clinical scenarios.

### Distraction

VR can be a method of distraction for overcoming uncomfortable medical procedures or as a nonpharmaceutical therapy for pain management. Current regimens in pain management, including surgical treatment, physical rehabilitation, or implantable drug-delivery systems, can be costly and ineffective [19]. Furthermore, opioids prescribed for pain control may lead to dependency and misuse [19]. Studies reporting positive results of VR interventions for pain management suggest that nurses working in a variety of care settings could use VR as an alternative therapy for pain relief [11]. In a randomized controlled study with 120 adults hospitalized in orthopedics or internal medicine with a pain score of at least 3 out of 10, those using multiple options of VR experience (eg, meditation, game, and nature experience) through a headset showed significant reductions in pain after 48 and 72 hours compared with those watching a television channel on health and wellness [8]. Importantly, the effect of this VR intervention was more pronounced in patients with severe baseline pain (pain score 7 out of 10) [8]. VR can also assist patients in coping with uncomfortable and even painful medical procedures [2]. Piskorz et al [20] found that 36 children whose attentions were distracted by playing or watching VR games during blood sampling reported significantly lower pain and stress than 21 children who did not experience VR.

### Relaxation and Mindfulness

VR can be an effective and feasible relaxation tool for individuals suffering from stress and psychological symptoms [5]. One experimental study assessed the effects of a VR intervention that provided relaxing videos of nature along with guided meditation and muscle relaxation to 49 individuals with depression, anxiety, or bipolar disorder [14]. This study revealed the beneficial effects of the VR intervention in improving mood and decreasing symptoms of depression and anxiety [14]. Kamińska et al [12] tested a 15-minute nature-based relaxation training program that comprised forest scenery and birdsong to relieve workplace stress in 28 office workers. The participants experienced significant improvements in perceived stress and mood [12]. Nurses are subject to workplace stress and symptoms such as anxiety and depression, and the recent pandemic has further aggravated the problem [21]. As a result, some hospitals have implemented VR experiences for frontline health care workers, explaining that VR may help them detach from stressful work environments and feel relaxed [22].

VR has the potential to increase mindfulness, focusing one’s attention on the present moment with an accepting and nonjudgmental attitude [23]. Developing mindfulness can reduce stress and improve symptoms, but mindfulness can be difficult to practice due to environmental and personal distractions [23]. VR may address these difficulties by providing an immersive virtual environment that supports the user to focus on the present moment. Seabrook et al [13] found that a VR mindfulness app that delivered a 15-minute program of a peaceful forest scenery video with a guided mindfulness voice-over significantly increased mindfulness and positive emotions in 37 adults. The participants reported a strong sense of being in the virtual forest while using the program [13]. Studies examining VR use to enhance relaxation and mindfulness have often involved healthy populations [13,15,23], yet increasing levels of mindfulness using VR may be beneficial to patients with a variety of physical and mental conditions.

### Cognitive Coping

The potential of VR-based therapy is gaining attention as a way to develop cognitive coping skills in individuals with psychiatric disorders such as posttraumatic stress disorder (PTSD) and depressive disorder. Peskin et al [16] tested 12 weekly 90-minute VR exposure therapy sessions in 25 adults with PTSD following the September 11 attacks. The participants described their trauma in detail while being exposed to VR scenarios simulating the attacks and reported significant decreases in PTSD, which in turn led to decreased depressive symptoms [16]. Another experimental study assessed the effects of up to 16 sessions of VR-based cognitive behavioral therapy (CBT) in 15 adult patients diagnosed with generalized social anxiety disorder [17]. The participants were exposed to behaviors and sounds of digital humans in virtual environments that simulated places that might trigger anxiety for these individuals, such as crowded cafés or supermarkets. This study indicated that VR-based CBT can be effective in improving social anxiety and depressive symptoms [17]. These studies suggest that VR may be a useful adjunct to psychotherapy to safely expose individuals to their traumatic triggers in a carefully controlled environment, enabling them to establish healthy cognitive coping skills over time.

### Rehabilitation

In recent years, VR has shown its potential as an assistive technology to aid the rehabilitation of patients with impaired neuromotor functions. Although VR cannot replace conventional physical or occupational therapies, it can promote the effectiveness of rehabilitation by providing task-oriented and multisensory training within an individualized safe virtual environment [18]. Mekbib et al [24] developed a VR-based rehabilitation system and validated its therapeutic potential in 23 recent patients who had stroke. The system provided upper extremity training in an immersive virtual environment and was effective in recovering upper extremity motor function when combined with conventional occupational therapy [24]. Regularity and repetition are essential in rehabilitation training for individuals with neurological disorders; however, motivation and adherence often decrease over time [18]. VR combined with traditional rehabilitation training may improve motivation and training outcomes. Winter et al [25] demonstrated that VR-based treadmill training improved motivation and gait rehabilitation in 36 healthy participants and 14 patients with multiple sclerosis or stroke. VR-assisted therapy is also promising for rehabilitation of children with neuromotor impairments. In the study by Bortone et al [26], VR games were introduced to 8 children with cerebral palsy or developmental coordination disorder through immersive virtual environments and wearable haptic devices, improving the children’s functionality of upper extremity.

## Future Implications for Practice and Research in Nursing

As VR technology becomes more popular and diverse in contents, the application of VR is expanding beyond education and training to support treatments or therapies and to promote wellness. Nurses can incorporate VR into clinical practice to improve treatment experience of patients and caregivers. Nurses may introduce relaxing VR experiences to reduce stress and anxiety in patients undergoing uncomfortable and painful procedures or surgeries [27]. VR can be a supportive medium to alleviate distress of patients undergoing intense treatments, such as chemotherapy [28]. Entertaining elements of VR can be effective in relieving pain and promoting psychological well-being in hospitalized patients, especially pediatric patients who tend to be more interested in gaming technology [29]. For patients with impaired cognitive or neuromotor functions, VR-based programs may stimulate their interests and motivate them to engage in long-term therapy sessions or rehabilitation [17,25].

For caregivers as well as patients, VR could also be useful for coping with stress and improving symptoms. While caring for individuals with acute or chronic conditions, caregivers often experience high levels of stress and associated multiple symptoms (eg, fatigue, sleep disturbance, depression, anxiety, and impaired cognition) due to the burden of caregiving tasks and changes in their circumstances [30]. Caregivers tend to be reluctant to leave the patient to practice their own health-promoting activities [31], but limited resources are available that enable caregivers to cope with stress without leaving the side of the patient. Through a VR platform, caregivers could briefly escape their real circumstances by exploring peaceful nature scenes or practicing mindfulness, which may lead to stress reduction and symptom improvement.

Although VR is a promising technology that can be used in diverse care settings, the realities we face in our practice and research may differ from our expectations. While some patients and caregivers are interested in and willing to use VR, others may be skeptical about the technology [8]. They may think that using VR takes away therapeutic encounters with care providers, and those with less affinity for technology may be somewhat averse to trying VR [32]. As such, the acceptance of VR depends on each person’s characteristics and perception and attitudes toward VR use [32]. More evidence should be generated regarding factors closely associated with VR acceptance. To optimize the opportunities VR can provide in health care, nurses need to first become familiar with the technology so that they can introduce and educate patients and caregivers about VR-based programs. It is important to create an environment that supports nurses to be competent in using digital health technologies, including VR [33]. Efforts also should be made to evaluate and improve user-friendliness and usefulness of VR-based programs perceived by various users [34].

As for research, more large-scale, long-term studies are needed to rigorously evaluate the effects of VR interventions on mood, cognitive function, as well as physical and psychological symptoms across different age groups and clinical populations. Expanding scientific research of physiological markers (eg, pulse rate, cortisol, cytokines, and genomic DNA) or neuroscientific measures (eg, real time functional magnetic resonance imaging) will also contribute to understanding the impact of VR interventions [35,36]. In addition, research on VR use in care settings should be practical, examining not just whether the interventions are effective, but also whether they are feasible for nurses to use in their practice and satisfactory to subjects [37]. Another important consideration when it comes to VR research is designing the research based on theoretical knowledge. Designing VR research referring to well-validated theories helps develop interventions that reflect the characteristics and needs of the target population; however, existing VR studies have rarely discussed theoretical components [34]. Further efforts are encouraged to develop and perform clinically meaningful and methodologically robust theory-based VR research.

When introducing VR to real subjects, especially patients, we should always keep in mind that we need to ensure their safety from any potential side effects. Nausea and dizziness caused by simulation sickness have been reported as common side effects associated with VR experiences [38]. VR-related side effects could lead to physical risks or injuries in those who are sensitive to motion sickness or individuals with impaired functions [39]. To prevent these potential side effects, it is recommended that VR interventions be applied for a limited period of time in a quiet and safe place to individuals who do not have restrictions on using the technology [37]. Nurses and other staff members also need to assess any physical discomfort associated with wearing VR devices, especially a bulky head-mounted display [37].

We should assess cost-effectiveness as well as efficacy and safety in order to leverage VR technology in clinical practice. VR is a high-cost technology that requires software development and equipment [34]. Implementing VR-based programs without considering cost-effectiveness within the already expensive US health care system could increase care costs and worsen health disparities caused by an individual’s ability to pay for such novel programs [40]. People with lower education and income levels may lag in getting information on treatments using VR technology as they tend to have limited eHealth literacy and poor access to health resources [41]. Care should be taken to ensure that all individuals have equitable access to participation in VR-based treatments and interventions.

Textbox 1 summarizes recommendations when incorporating VR into nursing practice and research.

**Summary of recommendations for use of virtual reality in clinical practice and research.**

- Research recommendations
  Investigate factors associated with virtual reality acceptance
  Assess user-friendliness and usefulness of virtual reality interventions
  Evaluate the effectiveness of virtual reality interventions in a variety of populations (eg, pediatric patients and caregivers)
  Establish virtual reality interventions based on theoretical components
  Conduct long-term studies with large samples
  Use physiological and neuroscientific measures as well as self-reported measures
  Assess nurses’ perception whether virtual reality interventions are feasible in practice
  Collect data regarding the optimal frequency, duration, and timing of use for virtual reality interventions
- Implications for clinical practice
  Create an environment that supports nurses becoming familiar with virtual reality
  Ensure participant safety from potential virtual reality-related adverse effects or discomfort
  Assess cost-effectiveness of virtual reality in the current health care system
  Establish evidence-based guidelines for implementing virtual reality interventions

## Conclusions

With care recipient needs and care environments becoming more complex, VR technology may provide opportunities to improve clinical practice and research. Beyond health care staff and students’ education and training, evidence in the literature supports VR use in pain management, distraction from difficult medical procedures and treatments, relaxation and mindfulness, CBT, and rehabilitation. Future studies are encouraged to evaluate how the impact of VR use differs across age groups and populations and explore which VR interventions are appropriate for each group and population. Evidence-based practice guidelines for implementing VR in care settings should be established through active research and quality improvement activities. Nurses need to be equipped with up-to-date information on VR use in health care, including trends and prospects, and interact with experts in relevant fields. We cannot stop the flow of novel technologies into health care. The use of such technologies should be optimized in a way that supports the current health care delivery.

## Abbreviations

CBT: cognitive behavioral therapy
PTSD: posttraumatic stress disorder
VR: virtual reality
